# Characterization of a Long-Lived Alginate Lyase Derived from *Shewanella* Species YH1

**DOI:** 10.3390/md16010004

**Published:** 2017-12-27

**Authors:** Hisashi Yagi, Natsuki Isobe, Narumi Itabashi, Asako Fujise, Takashi Ohshiro

**Affiliations:** 1 Center for Research on Green Sustainable Chemistry, Tottori University, 4-101 Koyamacho-minami, Tottori-city 680-8552, Tottori Prefecture, Japan; 2 Department of Biotechnology, Faculty of Engineering, Tottori University, 4-101 Koyamacho-minami, Tottori-city 680-8552, Tottori Prefecture, Japan; nt14.qi8@gmail.com (N.Is.); akrxxxrm.gern@gmail.com (N.It.); 3 Department of Chemistry and Biotechnology, Graduate School of Engineering, Tottori University, 4-101 Koyamacho-minami, Tottori-city 680-8552, Tottori Prefecture, Japan; fujiseasako@gmail.com (A.F.); oshiro@bio.tottori-u.ac.jp (T.O.)

**Keywords:** alginate lyase, *Shewanella* sp., PL family 7, characterization, alginate

## Abstract

Polysaccharides from seaweeds are widely used in various fields, including the food, biomedical material, cosmetic, and biofuel industries. Alginate, which is a major polysaccharide in brown algae, and the products of its degradation (oligosaccharides) have been used in stabilizers, thickeners, and gelling agents, especially in the food industry. Discovering novel alginate lyases with unique characteristics for the efficient production of oligosaccharides may be relevant for the food and pharmaceutical fields. In this study, we identified a unique alginate lyase derived from an alginate-utilizing bacterium, *Shewanella* species YH1. The recombinant enzyme (rAlgSV1-PL7) was produced in an *Escherichia coli* system and it was classified in the Polysaccharide Lyase family 7. The optimal temperature and pH for rAlgSV1-PL7 activity were around 45 °C and 8, respectively. Interestingly, we observed that rAlgSV1-PL7 retained over 80% of its enzyme activity after incubation at 30 °C for at least 20 days, indicating that rAlgSV1-PL7 is a long-lived enzyme. Moreover, the degradation of alginate by rAlgSV1-PL7 produced one to four sugars because of the broad substrate specificity of this enzyme. Our findings suggest that rAlgSV1-PL7 may represent a new commercially useful enzyme.

## 1. Introduction

The application of algal biomass is expected to increase in various fields, including the commercial food and pharmaceutical industries [1,2,3]. Alginate, which is a linear acidic polysaccharide, is located in the intercellular matrix of brown seaweed, representing about 30% of the dry weight of these seaweeds [4]. Additionally, it is currently used as an additive (i.e., as a stabilizer, viscosifier, and gelling agent) for food processing. The constituent sugar of alginate is β-d-mannuronic acid (M) linked to its C-5 epimer α-l-guluronic acid (G) with 1,4-*O*-glycoside bonds. Therefore, alginate consists of the following three blocks: two homopolymeric blocks [polyguluronic acid (polyG) and polymannuronic acid (polyM)] and one heteropolymeric block [random region (polyMG)] [4,5].

Various oligosaccharides derived from alginate via the degradative enzymes have physiological functions and biological activities (i.e., plant growth-promoting, antioxidant, anticoagulant, antitumor, antihypersensitive, antiproliferative, and anti-allergy activities) [6,7,8,9,10,11,12,13,14]. However, acid-hydrolyzed alginate tends to be less active than enzymatically degraded alginate [7,15]. Thus, discovering novel alginate-degrading enzymes or modifying the activities of known enzymes may have important implications for the food and pharmaceutical industries.

Alginate lyases belong to Polysaccharide Lyase (PL) families 5–7, 14, 15, 17, and 18 [16]. According to their substrate specificity, alginate lyases are mainly classified as either polyG lyases (EC 4.2.2.11) (polyG- and polyMG-specific) or polyM lyases (EC 4.2.2.3) (polyM-specific). Moreover, these enzymes function in an exolytic (PL-6, 15, and 17) or endolytic (PL-5 and 7) manner, with the β-elimination of the 4-*O*-glycosidic bonds accompanied by the formation of a double bond between C-4 and C-5 [17,18]. The degradation of alginate by various alginate lyases associated with extra- and intracellular degradation pathways involves several steps. First, PL-5 and -7 alginate lyases endolytically degrade alginate to produce oligosaccharides. Next, PL-6, -15, and -17 oligoalginate lyases exolytically degrade the oligosaccharides to form monosaccharides [18]. Finally, the monosaccharides are converted to 4-deoxy-l-erythro-5-hexoseulose uronic acid in a nonenzymatic reaction for use by the metabolic system.

Although an Enzyme Commission (EC) number has not been assigned, some alginate lyases were recently observed to be active against both polyG and polyM. Thus, these enzymes degrade alginate to produce oligosaccharides more effectively than other enzymes with similar functions [19,20,21]. Additionally, some alginate lyases are tolerant to heat, cold, and salt stresses [22,23,24]. These characteristics are important for enzymes used in the commercial production of oligosaccharides. Furthermore, enzyme stability (i.e., long-lived activity) is also a key consideration, particularly for industrial applications, because long-lived enzymes can help to decrease the input costs related to oligosaccharide production.

In this study, we focused on marine bacterial alginate lyases useful for industrial applications. We cloned and expressed a unique alginate lyase (AlgSV1-PL7) derived from *Shewanella* species YH1. Although the recombinant enzyme (rAlgSV1-PL7) contained a conserved QVH sequence, which is thought to indicate polyM specificity, it could use polyG and polyMG, as well as polyM as substrates [25,26]. More interestingly, rAlgSV1-PL7 retained 80% of its activity after incubation at 30 °C for at least 20 days. Additionally, rAlgSV1-PL7 produced one to four sugars from alginate. Our findings suggest that a long-lived rAlgSV1-PL7 may be useful as an industrial enzyme.

## 2. Results

### 2.1. Cloning and Purification of rAlgSV1-PL7

**Figure 1:**
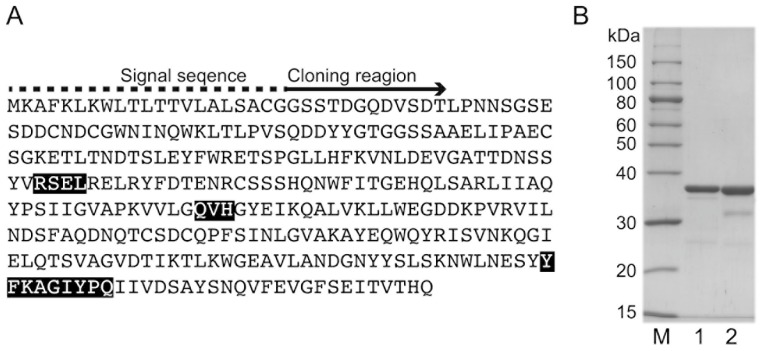
SDS-PAGE analysis of rAlgSV1-PL7 and determination of the amino acid sequence. (**A**) rAlgSV1-PL7 amino acid sequence based on an analysis of a draft genome. The rAlgSV1-PL7 signal sequence (dashed line) analyzed using the SignalP Server indicated that the enzyme is localized in the periplasm. The N-terminal region after removal of the signal sequence is indicated (arrow). The rAlgSV1-PL7 enzyme includes three conserved amino acid sequences (RSEL, QVH, and YFKAGIYPQ), indicating that it belongs to the PL7 family. (**B**) SDS-PAGE analysis of rAlgSV1-PL7. Lane M, molecular weight markers; Lane 1, purified rAlgSI-PL7 as a control [27]; Lane 2, purified rAlgSV1-PL7.

A BLAST search was conducted using an amino acid sequence (i.e., query) obtained by analyzing a draft genome (https://blast.ncbi.nlm.nih.gov). The search results indicated the target alginate-degrading enzyme belongs to the PL family 7 (Figure 1A). Moreover, it contains the conserved sequence QVH. Thus, we named the enzyme AlgSV1-PL7. An analysis using the SignalP 4.1 Server (http://www.cbs.dtu.dk/services/SignalP/) revealed that AlgSV1-PL7 contains a signal peptide, suggesting it is localized in the periplasm. We cloned rAlgSV1-PL7 lacking the signal peptide in an *Escherichia coli* expression system. The rAlgSV1-PL7 in *E. coli* BL21 cells was present in inclusion bodies, whereas 50% of the rAlgSV1-PL7 in *E. coli* SHuffle cells was in the soluble fraction. Additionally, *E. coli* SHuffle cells induced disulfide bonds in their expression system. Therefore, we purified the rAlgSV1-PL7 expressed in the *E. coli* SHuffle cells. An SDS-PAGE confirmed the purification of rAlgSV1-PL7 (Figure 1B, lane 2). Another *Shewanella* sp. YH1 alginate lyase, rAlgSI-PL7, was used as a control for the SDS-PAGE (Figure 1B, lane 1) because its theoretical molecular weight was similar to that of rAlgSV1-PL7. The theoretical pI and molecular weight of rAlgSV-PL7 without the signal peptide were 4.6 and 33,216.1 Da, respectively.

### 2.2. Determining the Optimal Temperature and pH for rAlgSV1-PL7 Activity

**Figure 2:**
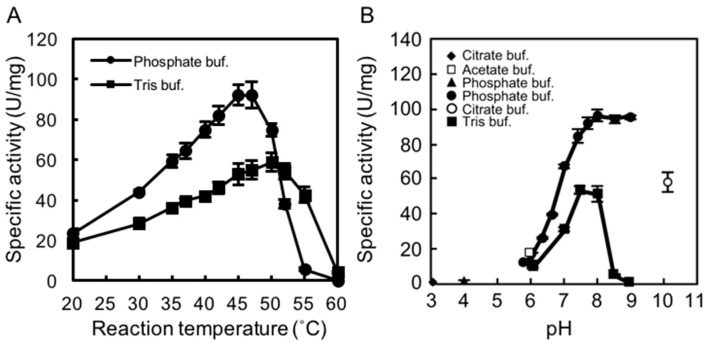

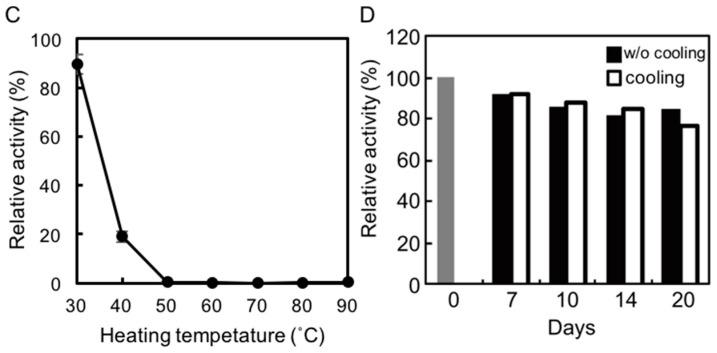
Effects of temperature and pH on rAlgSV1-PL7 activity. (**A**) Optimal temperature for rAlgSV1-PL7 activity. Lyase activity was measured in Tris-HCl and potassium phosphate buffers. (**B**) Optimal pH for rAlgSV1-PL7 activity. Lyase activity was measured for rAlgSV1-PL7 in citrate buffer (pH 3, 4 and 10), sodium acetate buffer (pH 6), potassium phosphate buffer (pH 5.7–9), and Tris buffer (pH 6–9). (**C**) Thermostability of rAlgSV1-PL7. The samples were incubated at each temperature for 1 h and then cooled on ice. (**D**) A long-lived activity assay of rAlgSV1-PL7 at 30 °C. The samples were measured with (white bars) or without (black bars) a cooling step. The control (0 day) was shown in grey bar. The enzyme activity assays (**C**,**D**) were performed with samples prepared in Tris buffer.

We examined the effects of temperature and pH on rAlgSV1-PL7 activity. A lyase activity assay with sodium alginate (1000 cps) as the substrate was used to calculate the specific activity. To determine the optimal temperature for rAlgSV1-PL7 activity, we examined the effects of Tris and phosphate buffers at temperatures between 20 and 60 °C. Interestingly, the enzymatic activity was about two times higher in the phosphate buffer than in Tris buffer, with an optimal temperature of around 45 °C (Figure 2A), which was consistent with the results of an earlier study involving AlgSI-PL7 [27]. Moreover, although the optimal pH for rAlgSV1-PL7 activity in the phosphate buffer was around 8.0, the enzyme retained its lyase activity between pH 7.5 and 9 (Figure 2B). The lyase activity in Tris buffer decreased when the pH exceeded 8.5. These results suggested that optimal rAlgSV1-PL7 activity was generally achieved under neutral conditions.

### 2.3. Thermostability of rAlgSV1-PL7

To assess the thermostability of rAlgSV1-PL7, the enzyme was incubated in Tris buffer at various temperatures for 1 h, after which lyase activity was measured (Figure 2C). Approximately 90% of the rAlgSV1-PL7 activity was retained at 30 °C, while only about 20% of the activity was retained at 40 °C. Lyase activity was undetectable at temperatures greater than 50 °C, implying that rAlgSV1-PL7 is not a thermotolerant enzyme. However, because rAlgSV1-PL7 was highly active at 30 °C, we examined whether the activity was long-lived (Figure 2D). To assess the refolding of rAlgSV1-PL7, we analyzed the samples with and without a cooling step. Approximately 80% of the enzyme activity was retained after 20 days regardless of whether the samples were cooled before measurements, implying that the structure of rAlgSV1-PL7 was highly stable at 30 °C to ensure long-lived activity.

### 2.4. Effects of Metals and Inhibitors on rAlgSV1-PL7

**Figure 3:**
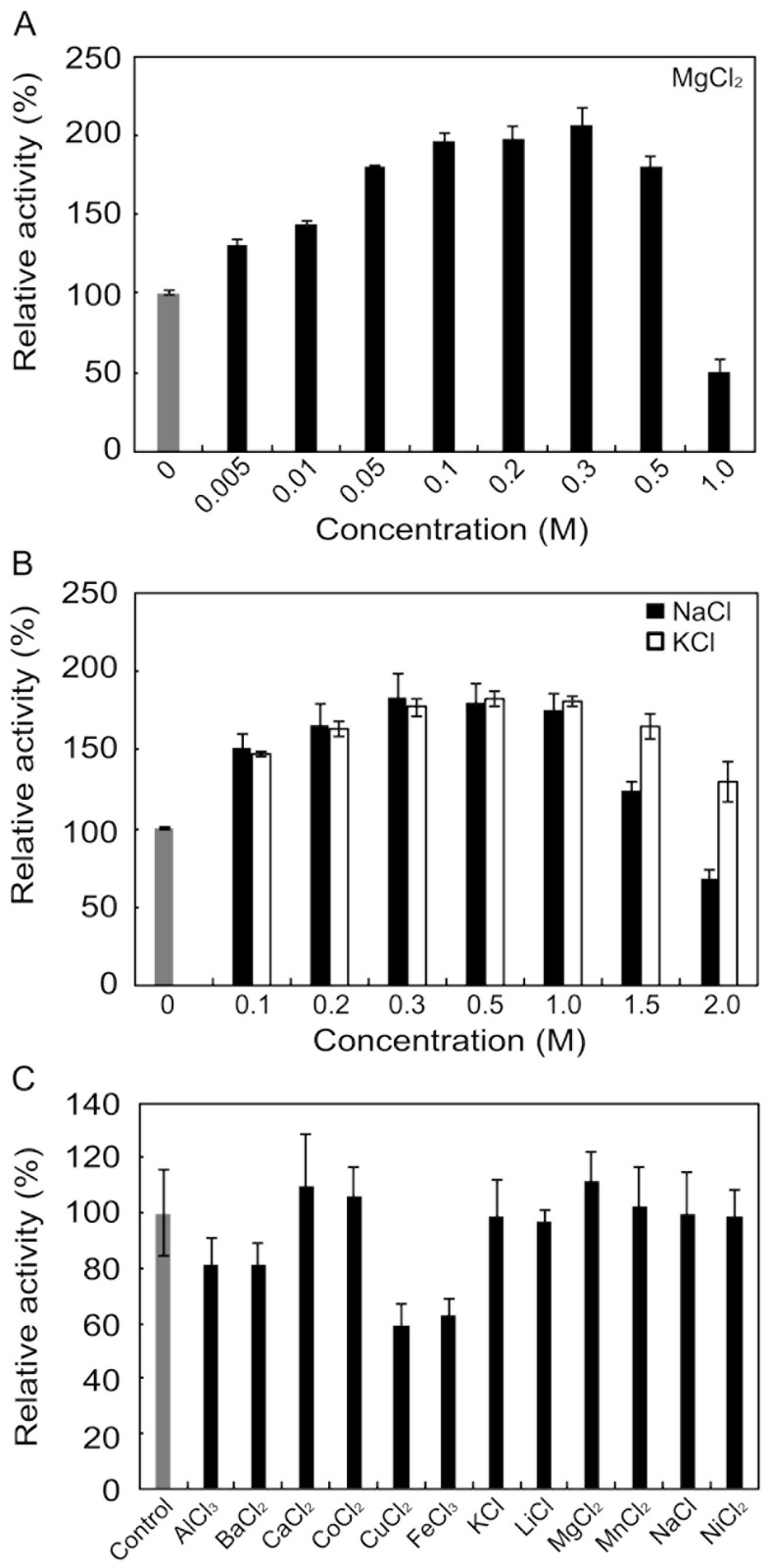
Effects of metals on rAlgSV1-PL7 activity. Effects of MgCl_2_ (**A**), NaCl (black), and KCl (white) (**B**) on rAlgSV1-PL7 lyase activity. (**C**) Effects of various metals (1 mM) on rAlgSV1-PL7 lyase activity. The activities are presented relative to the activity in the absence of metals. Each control was shown in grey bars.

Although many alginate lyases are activated or inactivated by various metals and inhibitors, Na^+^, K^+^, and Mg^2+^ tend to function as activators. We examined the concentration-dependent effects of these metal ions on rAlgSV1-PL7 (Figure 3A,B). The enzyme activity was nearly 2-fold following treatments with 0.3 M Mg^2+^, 0.8 M Na^+^, or K^+^. However, increasing the Mg^2+^ concentration to 1 M resulted in a decrease in the enzyme activity to 50%. In contrast, rAlgSV1-PL7 activity remained relatively high at 1.5 M Na^+^ or K^+^. Moreover, the enzyme activity was still approximately 30% higher in the presence of 2 M K^+^ than in the absence of K^+^, implying that rAlgSV1-PL7 is a salt-tolerant enzyme. We also examined the effects of various metals (1 mM) and observed no major decreases in rAlgSV1-PL7 activity (Figure 3C). Finally, we investigated the effects of inhibitors on rAlgSV1-PL7 (Table 1). The enzyme activity was unaffected by all the tested compounds, with the exception of *N*-bromosuccinimide, which completely inhibited rAlgSV1-PL7 activity. The fact that EDTA did not affect rAlgSV1-PL7 suggested that this enzyme does not require metal cofactors. These results indicated that rAlgSV1-PL7 may be a manageable and useful enzyme for industrial applications.

### 2.5. Substrate specificity of rAlgSV1-PL7

**Figure 4:**
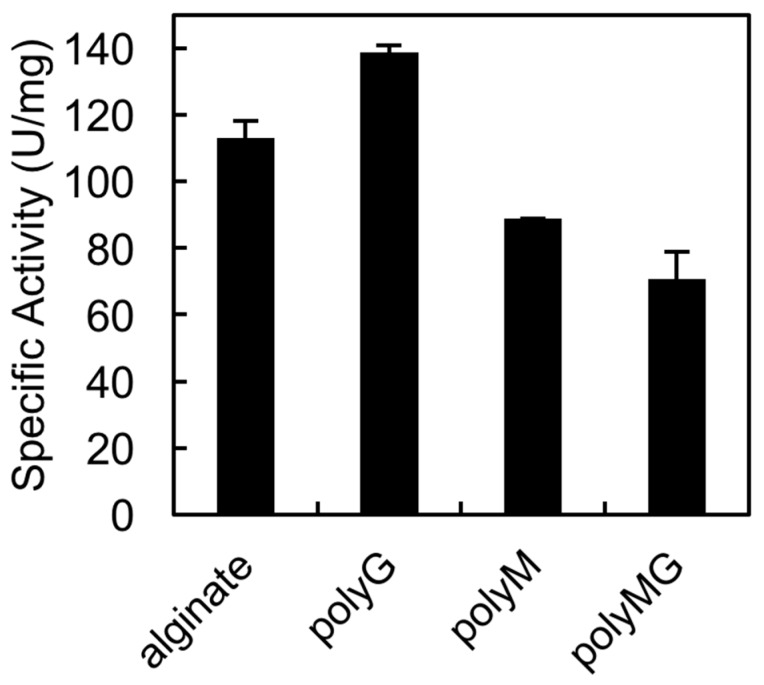
Substrate specificity of rAlgSV1-PL7. Substrates were sodium alginate (1000 cps) and the synthesized blocks. PolyMG, polyG, and polyM were prepared as described by Haug et al.

The QIH and QVH sequences, which are conserved among PL7 family members, are important for substrate recognition. Previous studies confirmed that QIH is specific for polyG and polyMG, while QVH is specific for polyM [25,26]. In an earlier study, we proved that AlgSI-PL7 from *Shewanella* sp. YH1, which contains the QIH sequence, is a polyG-specific enzyme [27]. In the current study, we tested whether rAlgSV1-PL7 is a polyM-specific enzyme (Figure 4). We observed that rAlgSV1-PL7 was highly active against polyG, but could also use polyM and polyMG as substrates. Therefore, rAlgSV1-PL7 appears to exhibit a broad substrate specificity.

### 2.6. Detection of the Products Resulting from the Degradation of Sodium Alginate by rAlgSV1-PL7

**Figure 5:**
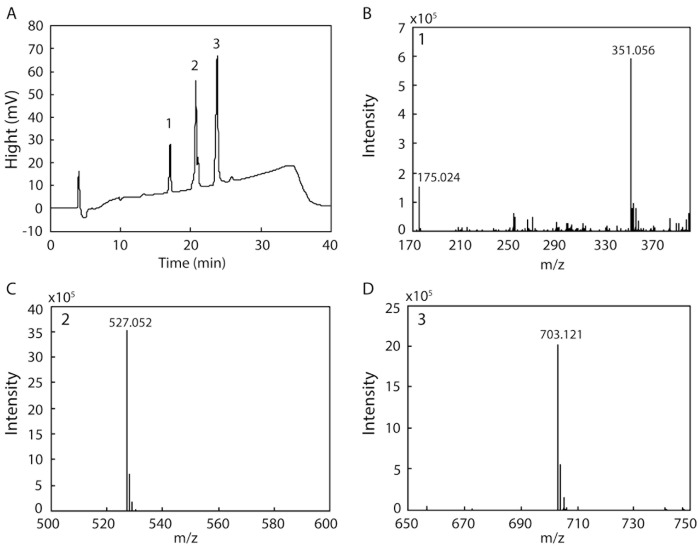
HPLC and electrospray ionization mass spectrometry (ESI-MS) analyses of the products generated from the degradation of alginate by rAlgSV1-PL7. (**A**) HPLC analysis of the degradation products (1: 17 min (**B**), 2: 20 min (**C**) and 3: 23 min (**D**)). (**B**) The 175 and 351 peaks corresponded to mono- and disaccharides derived from alginate [28]. (**C**,**D**) Additional oligosaccharides produced from the degradation of alginate (527.052 = trisaccharides; 703.121 = tetrasaccharides). The mass spectrometer was operated in the negative mode.

Several endo- and exotypes of alginate lyases (oligoalginate lyases) are typically involved in the degradation of alginate to monosaccharides. However, some alginate lyases can degrade alginate to mono-, di-, and trisaccharides by themselves. We examined the products resulting from the degradation of alginate by rAlgSV1-PL7 because of the broad substrate specificity of this enzyme. After a 48 h incubation, degraded fractions collected by HPLC were analyzed by electrospray ionization mass spectrometry (ESI-MS) (Figure 5). The result of the HPLC analysis indicated that we obtained three peaks (1: 17 min, 2: 20 min, 3: 23 min) (Figure 5A). Each peak was then used to determine the degradation products by ESI-MS. We detected four major peaks corresponding to a monosaccharide (Figure 5B: 175.024), disaccharide (Figure 5B: 351.056), trisaccharide (Figure 5C: 527.052), and tetrasaccharide (Figure 5D: 703.121), with *m*/*z* values that were consistent with those of previous reports [28,29,30]. Although the main degradation products were tri- and tetrasaccharides, we also detected mono- and disaccharides. Therefore, the degradation of alginate by rAlgSV1-PL7 results in the production of one to four oligosaccharides.

## 3. Discussion

### 3.1. Analysis of a Draft Genome to Identify Shewanella sp. YH1 Alginate Lyases

Alginate lyases are polysaccharide lyases that have been well studied. Although the alginate lyase genes have been identified in many alginate-utilizing bacteria, there are relatively few reports describing the alginate lyases of *Shewanella* spp. [18,25,31,32,33,34,35,36]. However, *Shewanella* sp. PL-6 and -17 oligoalginate lyases were recently described [33,34]. An analysis of a draft genome combined with a BLAST search indicated that *Shewanella* sp. YH1 contains two PL-6 enzyme genes, three PL-7 enzyme genes, two PL-17 enzyme genes, and one unclassified enzyme gene [27]. A homology search revealed that the alginate lyase with a sequence most similar to that of rAlgSV1-PL7 (58%) was derived from *Shewanella* sp. UCD-KL21 (accession WP 076541881), implying that rAlgSV1-PL7 is a newly discovered *Shewanella* sp. alginate lyase. We previously characterized a *Shewanella* sp. YH1 PL-7 lyase, AlgSI-PL7, which contains the conserved QIH sequence [27]. In this study, we characterized another PL-7 lyase, rAlgSV1-PL7. Because both of these enzymes exhibit unique characteristics, *Shewanella* sp. YH1 may have several unique enzymes. Thus, we will characterize additional alginate lyases in future studies to more thoroughly elucidate the mechanisms regulating the degradation of alginate by *Shewanella* sp. YH1.

### 3.2. Enzymatic Characteristics of rAlgSV1-PL7

We tested various buffers to determine the optimal conditions for rAlgSV1-PL7 activity (Figure 2A,B). We observed that rAlgSV1-PL7 lyase activity was enhanced in phosphate buffer, which was consistent with the results of an earlier study involving AlgSI-PL7 [27]. Thus, *Shewanella* sp. YH1 enzymes appear to prefer phosphate buffers, although the mechanisms underlying this preference remain unclear. Additionally, the activities of alginate lyases derived from *Pseudoalteromonas* sp. strain No. 272 and *Turbo cornutus* reportedly decrease in Tris buffer [37,38]. The amino group of Tris might adversely affect the stability of rAlgSV1-PL7 through electrostatic interactions. 

We observed that rAlgSV1-PL7 remained active in the presence of 1.0–2.0 M NaCl and KCl, suggesting that rAlgSV1-PL7 is tolerant to salt stress. Salt-tolerant enzymes generally have relatively high acidic amino acid contents. For example, the alginate lyases of halophilic Gram-negative bacteria have an abundance of Asp and Glu residues [e.g., *Halomonas* sp. Victoria JH alginate lyase (GenBank accession number: ANB32492) (16.1%) and *Cobetia crustatorum* alginate lyase (NCBI reference sequence: WP_024952190) (15.9%)]. Therefore, the 16% Asp and Glu content may be related to the salt-tolerance of alginate lyases. Although the 13% Asp and Glu content calculated for rAlgSV1-PL7 was below the percentage for known salt-tolerant alginate lyases, it was higher than the 11% reported for non-salt-tolerant alginate lyases. 

Interestingly, rAlgSV1-PL7 retained its activity for at least 20 days (Figure 2D) with or without a cooling step after incubation at 30 °C. Enzymes that are partially unfolded at 30 °C may refold to their functional state after a cooling treatment. However, the lack of any difference between the activities of the cooled and noncooled rAlgSV1-PL7 suggests that this enzyme is structurally stable in mild conditions. The rAlgSV1-PL7 amino acid sequence includes six cysteine residues. Our size exclusion chromatography results indicated that rAlgSV1-PL7 was eluted as a monomer. Therefore, it is probable that intramolecular disulfide bonds had formed, although the exact pairing of cysteine residues is unclear. Furthermore, we previously determined that AlgSI-PL7 from *Shewanella* sp. YH1 and AlgC-PL7 from *Cobetia* sp. NAP1 contain two cysteine residues in the C-terminal region [23]. The disulfide bond in these enzymes may not affect their structural stability. In contrast, if the six cysteine residues of rAlgSV1-PL7 form intramolecular disulfide bonds, they regulate the conformation of the enzyme and may contribute to the observed long-lived enzyme activity.

Cysteine residues may also influence enzyme thermostability. Some bacteria isolated from deep-sea hydrothermal vents, such as *Nitratiruptor* sp. SB155-2, *Caminibacter mediatlanticus*, *Hippea jasoniae*, and *Nautilia profundicola*, produce alginate lyases with cysteine residues around amino acid positions 80 and 232 [39]. These PL-7 alginate lyases exhibit thermostability because of an intramolecular disulfide bond. In contrast, the rAlgSV1-PL7 amino acid sequence does not contain cysteine residues near positions 80 and 232. Therefore, we will attempt to generate a mutated rAlgSV1-PL7 in which cysteine residues are inserted at these positions. We will then assess whether the additional cysteines can increase the long-lived activity and thermostability of the enzyme.

### 3.3. Effects of Metals and Inhibitors on rAlgSV1-PL7

Unlike the other tested metals, which did not affect rAlgSV1-PL7 activity, Cu^2+^ and Fe^3+^ decreased the enzyme activity by about 40% (Figure 3C). Additionally, rAlgSV1-PL7 activity was unaffected by a high EDTA concentration, indicating that rAlgSV1-PL7 may be activated by some metals, but metals are not essential for its activity. Moreover, previous studies revealed differences in the effects of inhibitors on polyG and polyM lyases [26,40]. We classified rAlgSV1-PL7 as a polyM lyase. Several polyM lyases are reportedly unaffected or only slightly activated by EDTA. Our results are consistent with those earlier studies. In the current study, exposure to *N*-bromosuccinimide, which is a tryptophan-modifying agent, resulted in the complete inactivation of rAlgSV1-PL7. Although rAlgSV1-PL7 includes eight tryptophan residues, two of them (positions 173 and 214) are located close to the catalytic region, suggesting they may be important for enzyme activity. Although rAlgSV1-PL7 has six cysteine residues, *N*-ethylmaleimide and DTNB did not affect the enzyme activity. The mechanisms responsible for these observations remain unclear. However, the active form of rAlgSV1-PL7 may be stable in the presence of these reagents.

### 3.4. Alginate Degradation by rAlgSV1-PL7

The conserved amino acid sequences in PL-7 lyases (QIH and QVH) are thought to determine substrate specificity (i.e., QVH: polyM-specific; QIH: polyG- and polyMG-specific) [25,26,41]. We previously revealed that *Shewanella* sp. YH1 AlgSI-PL7, which contains the QIH sequence, exhibits high specific activity against polyG and low specific activity against polyM. Although we expected rAlgSV1-PL7 to exhibit high specific activity against polyM, we observed that this enzyme exhibits high activity against polyG (Figure 4). Moreover, compared with AlgSI-PL7, rAlgSV1-PL7 exhibited higher specific activity against polyM and polyMG (i.e., a bifunctional activity). Although AlgC-PL7 is also bifunctional, its activities are lower than those of rAlgSV1-PL7, implying that rAlgSV1-PL7 may be a more commercially useful enzyme [23]. Regarding the degradation products, rAlgSV1-PL7 mainly produced low-molecular-weight oligosaccharides (di-, tri-, and tetrasaccharides) from alginate (Figure 5). These oligosaccharides reportedly have several bioactivities [42,43]. Furthermore, we detected a monosaccharide peak as well as AlgC-PL7 by ESI-MS. This was considered to be due to the substrate specificity (broad activity) of this enzyme. A combination of several alginate lyases is usually necessary to produce low-molecular-weight oligosaccharides, but our results indicate that rAlgSV1-PL7 can generate all of these degradation products.

In conclusion, we isolated and cloned a novel long-lived PL7 lyase (rAlgSV1-PL7) derived from *Shewanella* sp. YH1. We observed that rAlgSV1-PL7 is salt- and metal-tolerant and exhibits a high bifunctional activity to produce mono-, di-, tri-, and tetrasaccharides from alginate. These results suggest that rAlgSV1-PL7 is highly adaptable to environmental changes and may be a commercially useful enzyme.

## 4. Materials and Methods

### 4.1. Cloning and Expression of Recombinant AlgSV1-PL7

The *algsv1-pl7* gene was identified during the analysis of the *Shewanella* sp. YH1 genome using the HiSeq 2500 high-throughput sequencing system (Illumina Inc., San Diego, CA, USA) [23]. We designed polymerase chain reaction primers to amplify *algsv1-pl7* (forward primer 5′-GGAGATATACATATGGGTTCAAGTACTGATGG-3′; reverse primer 5′-GTGGTGGTGCTCGAGTTATTGATGTGTTACTGTTATC-3′). Additionally, *algsv1-pl7* was ligated into the pET-21a expression vector according to the In-Fusion HD cloning method (Takara Bio Inc., Shiga, Japan) for a subsequent transformation of *E. coli* HST08 premium competent cells (Takara Bio Inc.). To produce rAlgSV1-PL7, the purified pET-21a–*algsv1-pl7* vector was inserted into *E. coli* BL21 (DE3) or SHuffle cells, which were then cultured in LB broth containing ampicillin at 37 °C (BL21(DE3)) or 20 °C (SHuffle). We added 1 mM (final concentration) isopropyl β-d-1-thiogalactopyranoside (IPTG) to the bacterial culture to induce the production of rAlgSV1-PL7 lacking a signal peptide [23].

### 4.2. Purification of rAlgSV1-PL7

*E. coli* SHuffle cells were cultured in LB medium at 20 °C for 18 h with shaking (120 rpm). After 18 h, we added 1 mM (final concentration) IPTG to induce the production of rAlgSV-PL7 for 10 h. The cells were harvested and sonicated in 50 mM Tris-HCl buffer (pH 7.0). After centrifuging the lysed cells, streptomycin sulfate (2.5% final) was added to the supernatant to remove the nucleic acids. The sample was centrifuged and the supernatant was dialyzed in 50 mM *Tris*-HCl (pH 7.0) before being applied to a Resource Q column (GE Healthcare, Little Chalfont, UK). The samples were eluted with a linear gradient of 0–1 M NaCl. The collected protein fractions were applied to a Superdex 75 column (GE Healthcare). After dialysis, the samples were reapplied to a Resource Q column for a subsequent elution with a gradient of 90–130 mM NaCl. The concentration of the purified rAlgSV1-PL7 was determined using the Bradford method (Bio-Rad Laboratories, Hercules, CA, USA).

### 4.3. Enzymatic Activity Assay

A 1 ml reaction solution containing 0.1 M Tris-HCl buffer (pH 7.0), 0.2% (*v*/*v*) substrate [sodium alginate (1000 cps), polyMG, polyM, and polyG], and 5–10 µL (0.02–0.04 U) enzyme was prepared to analyze lyase activity. PolyMG, polyM, and polyG were prepared as previously described [5]. Lyase activity was measured at 42 °C for 3 min, and the absorbance at 232 nm was recorded. Experiments were performed to optimize the temperature (20–60 °C), pH (3–10; pH 3–4 and 10: citrate buffer; pH 6: sodium acetate buffer; pH 5.7–9: potassium phosphate buffer; and pH 6–9: Tris buffer), effects of metals and inhibitors (1 mM final concentration), and substrate specificity (polyG, polyM, and polyMG). For the thermostability experiment, the samples were preincubated at 30–90 °C for 1 h and then cooled on ice for at least 15 min. Additionally, long-lived enzyme activity was assessed by incubating the samples in a block heater set at 30 °C for 20 days, with or without a subsequent 30 min cooling period prior to measuring the enzyme activity. The samples were analyzed at 42 °C using a UV-2600 spectrophotometer (Shimadzu Corp., Kyoto, Japan). The absorbance at 232 nm increased by 5.5 when 1 mM unsaturated uronic acids was produced [44,45,46]. One unit of lyase activity was defined as the amount of enzyme required to produce 1 µmol oligosaccharides containing unsaturated uronic acids per minute.

### 4.4. Mass Spectrometry

The products resulting from the degradation of sodium alginate were examined by electrospray ionization mass spectrometry, with the resulting data analyzed by the Xcalibur program (Thermo Fisher Scientific, Waltham, MA, USA). The mass spectrometer was operated in the negative mode. To prepare the samples, the reaction mixtures were incubated at 30 °C for 48 h. After centrifugation, the samples were applied to a Superdex peptide column (GE Healthcare). The degradation products, rAlgSV1-PL7 and undigested sodium alginate, were separated based on the absorbance at 232 nm. The collected fractions were applied to an Amide-80 column (Tosoh, Tokyo, Japan) and the samples were eluted with a linear gradient of 95–70% acetonitrile.

## Figures and Tables

**Table 1:** Effects of potential inhibitors on rAlgSV1-PL7 activity.

Inhibitors	Concentration (mM)	Relative Activity (%)
Control	0	100.00 ± 2.68
2,2-bipyridine	1	93.71 ± 5.12
*N*-bromosuccinimide	1	0
*N*-ethylmaleimide	1	91.52 ± 1.32
EDTA	1	89.96 ± 6.14
DTNB	1	92.86 ± 2.46
DEPC	1	92.75 ± 3.49
